# The Pathology of Type 2 Inflammation-Associated Itch in Atopic Dermatitis

**DOI:** 10.3390/diagnostics11112090

**Published:** 2021-11-12

**Authors:** Catharina Sagita Moniaga, Mitsutoshi Tominaga, Kenji Takamori

**Affiliations:** 1 Juntendo Itch Research Center (JIRC), Institute for Environmental and Gender-Specific Medicine, Graduate School of Medicine, Juntendo University, 2-1-1 Tomioka, Urayasu 279-0021, Japan; m-catharina@juntendo.ac.jp (C.S.M.); ktakamor@juntendo.ac.jp (K.T.); 2 Anti-Aging Skin Research Laboratory, Graduate School of Medicine, Juntendo University, 2-1-1 Tomioka, Urayasu 279-0021, Japan; 3 Department of Dermatology, Juntendo University Urayasu Hospital, 2-1-1 Tomioka, Urayasu 279-0021, Japan

**Keywords:** atopic dermatitis, biologic agents, neuroimmune interactions, type 2 inflammation

## Abstract

Accumulated evidence on type 2 inflammation-associated itch in atopic dermatitis has recently been reported. Crosstalk between the immune and nervous systems (neuroimmune interactions) is prominent in atopic dermatitis research, particularly regarding itch and inflammation. A comprehensive understanding of bidirectional neuroimmune interactions will provide insights into the pathogenesis of itch and its treatment. There is currently no agreed cure for itch in atopic dermatitis; however, increasing numbers of novel and targeted biologic agents have potential for its management and are in the advanced stages of clinical trials. In this review, we summarize and discuss advances in our understanding of type 2 inflammation-associated itch and implications for its management and treatment in patients with atopic dermatitis.

## 1. Introduction

Atopic dermatitis (AD) is a common chronic inflammatory skin disorder with a complex pathophysiology and clinical heterogeneity in the age of its onset, morphology, and the distribution and severity of lesions [1,2]. The prevalence of AD is approximately 4% in adults and 10% in children, with 50% developing persistent skin disease as adults [3]. The pathophysiology of AD involves complex interactions between epidermal barrier disruption, skin microbiome dysbiosis, and altered type 2 immune responses [2,4].

One of the most common symptoms in dermatology clinics is itch, which is generally intractable despite the administration of medication [5]. Hawro et al. reported itch in 90% of patients with chronic skin diseases, and showed that itch intensity was associated with the disruption of sleep quality, work productivity, and mental health [6]. Several itch-related mediators and receptors are differently expressed in pruritic skin, suggesting an “itchscriptome” for each disease. As an example, AD and psoriasis with itch showed elevated gene transcript levels of interleukin (IL)-17A, IL-23A, and IL-31. However, the gene expression of transient receptor potential (TRP) vanilloid 2, TRP ankyrin 1, protease-activated receptor (PAR) 2, PAR 4, and IL-10 was up-regulated in pruritic AD skin only, while that of TRP melastatin 8, TRP vanilloid 3, phospholipase C, and IL-36a/g in psoriatic skin only. Specific “itchscriptomes” may provide a more detailed understanding of the molecular mechanisms underlying itch and its treatment targets [7].

Despite its heterogeneity, AD is generally managed by a “one-size-fits-all” therapeutic approach, rather than precise personalized, endotype, or ethnicity-driven therapeutic strategies [2,8]. A precise medical approach to the management of AD will rely on the discovery and validation of biomarkers that facilitate tailored management, including prevention strategies, and the treatment of patients with severe disease by targeted therapies [3,8]. In this review, we summarize the current status of the precision treatment of itch in patients with AD.

## 2. Disease Burden of AD

AD is ubiquitous with high morbidity and healthcare costs [4]. Moreover, it has a negative impact on the quality of life (QoL) of not only patients, but also their families and caregivers [9]. A cross-sectional study identified the most burdensome symptom as itch (54.4%), followed by excessive dryness/scaling (19.6%), and red/inflamed skin (7.2%). Severe itch has been associated with poor mental health [10]. Previous studies also revealed a correlation between suicidal ideation and AD in both girls and boys [11], which was also highly prevalent in patients with chronic pruritus [6]. Moreover, pruritus impairs sleep quality [12]. The pathophysiology of this impairment is complex and may involve inter-relatedness between sleep, the circadian rhythm, immune system, and environment [13].

Besides its psychosocial impact, AD causes major economic burdens [14], with the associated economic burden of severe AD being significant [15]. Luk et al. reported that the median annual cost of chronic pruritus was US$1067 per patient [16]. The economic burden of childhood AD in Australia, South Korea, and Singapore was USD 1000–6000 per patient annually [17]. Economic costs generally include both direct costs (e.g., the costs of medical visits, including tests, procedures, and medications) and indirect costs (e.g., the loss of earnings by patients or caregivers, productivity loss, informal caregiving, and transportation costs) [15]. Previous studies identified the most prominent costs as informal caregiving (46%) for childhood AD in Singapore [18], and productivity loss in AD patients receiving systemic immunosuppressive treatment [14]. 

## 3. Type 2 Inflammation and AD

Allergic diseases are mostly mediated by systemic type 2 helper T cell (Th2)–driven inflammation [19], which is characterized by CD4^+^ T cells and immunoglobulin E (IgE) of B cells. Type 2 immunity involves immune responses by innate and adaptive immune systems. Group 2 lymphoid cells (ILC2), eosinophils, basophils, mast cells, and IL-4- and/or IL-13-activated macrophages play roles in the innate immune system [20]. The activation of Th2 and ILC2 pathways may be at the core of type 2 inflammation, which involves IL-4, IL-5, IL-9, IL-13, and IL-31 as Th2 cytokines and IL-5, IL-9, and IL-13 as the essential type 2 cytokines of ILC2 [21,22]. 

The inflammatory cascade is triggered in response to allergens, leading to allergic diseases [20]. Although not limited to type 2 immune responses, epithelial-derived cytokines, e.g., thymic stromal lymphopoietin (TSLP), IL-25, and IL-33, play important roles in the stimulation and enhancement of type 2 responses. Upon exposure to allergens, infectious agents, and toxins, epithelial cells as the first line of defense, release alarmins, including TSLP, IL-25, and IL-33 [23,24], and may directly induce type 2 cytokine production by ILC2 [25].

ILC2 are a subgroup of ILCs, a unique subset of lymphocytes without rearranged antigen receptors. They are present in both humans and mice, produce type 2 cytokines, and may promote inflammation and hyperresponsiveness [26,27,28]. Kim et al. reported resident group ILC2 in healthy human skin that multiplied in AD skin lesions [29]. Moreover, skin-derived ILC2 were shown to express the IL-33 receptor ST2, which was up-regulated during activation, such as in an AD mouse model [30]. The IL-33-ILC2 axis has recently been proposed as the central mediator in human AD [31]. IL-33 induces IL-31 and may trigger pruritus and scratching bouts [31], suggesting a role for ILC2 in the pathogenesis of itch in AD. 

Evidence has been obtained that supports the systemic involvement of type 2 inflammation either in acute and chronic skin lesions or in the extrinsic and intrinsic classification of AD [32]. The initiation of acute lesions is accompanied by marked increases in antimicrobial peptide (AMP) levels (S100A7/S100A8/S100A9) and the up-regulation of Th2 and Th22 cytokines. The weaker induction of IL-17 was also observed in acute lesions. The intensification of the Th2 and Th22 cytokine axes with disease chronicity has been demonstrated, with significant increases being observed in Th1 markers in patients with chronic AD [33].

The circulating immune phenotype was defined in adults and young children with early AD. Czarnowicki et al. showed that a decreased Th1/Th2 ratio characterized the AD phenotype across all age groups, while IL-9, IL-22, and regulatory T cells were detected in patients other than infants. Differences in immune events between pediatric and adult AD patients suggest the need for age-specific, rather than uniform, therapeutic interventions [34].

Recent advances in our understanding of the pathophysiology of AD have implied that systemic type 2 inflammation is one of the underlying disease characteristics of AD, as evidenced by the activation of the Th2 pathway in the non-lesional skin of AD [35] as well as eosinophilia in the blood of AD patients [36]. Furthermore, the serum level of thymus and activation-regulated chemokine (TARC), an IL-4- and IL-13-induced chemokine that functions as a selective chemoattractant for T cells, was enhanced in AD patients compared with normal controls [37,38]. Furthermore, IL-4 has been shown to induce Th2 cell differentiation and isotype switching to IgE production in B cells [39], while IL-13 regulates the proliferation of IgE-producing B cells and disrupts the epithelial tight junction barrier [40,41]. 

In addition, a bacterial artificial chromosome (BAC) transgenic mouse model that overexpresses the type 2 cytokines, IL-4, IL-5, and IL-13, spontaneously developed AD-like skin lesions due to an exaggerated type 2 response, e.g., high serum IgE levels, excessive immune cell infiltration (including eosinophils and lymphocytes) in the skin, and dermal thickening [42]. 

Collectively, these findings demonstrate that AD is characterized by the potent activation of Th2 cells and ILC2, with the excessive production of type 2 cytokines, particularly IL-4 and IL-13. While the activation of type 2 immune responses is common in all patients with AD, the variable activation of epithelial-derived cytokines also disseminates this response [20,43]. 

## 4. Neuroimmune Interactions Associated with Type 2 Inflammation in Pruritic AD

Interactions between the nervous and immune systems are essential for sensing potential pathogens and activating protective mechanisms in the host [44,45]. Intensive crosstalk has been reported between these systems at multiple barrier surfaces, including the gut [46], lungs [47], and skin [48]. Various responses are induced by interactions involving neurophysiological reflexes, e.g., scratching to expel invading pathogens and noxious environmental stimuli [45,49].

**Figure 1:**
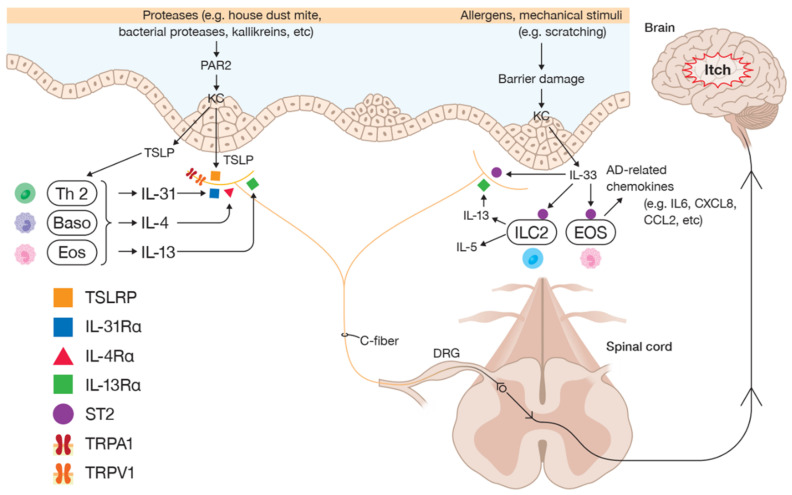
Neuroimmune crosstalk between keratinocytes, primary sensory neurons, and type 2 immune cells in AD skin. Epithelial barrier disruption during exposure to various allergens or triggers, e.g., proteases or scratching, induces keratinocytes to secrete alarmins, such as TSLP and IL-33. TSLP and IL-33 initiate allergic responses by activating ILC2, Th2 cells, and other immune cells, for the production of large amounts of type 2 cytokines, including IL-4, IL-5, IL-13, and IL-31. Alarmins and type 2 cytokines directly activate sensory neurons via their receptors, which signal to the somatosensory cortex in the brain triggering itch or itch sensitization in AD.

The itch–scratch cycle is a prominent feature of AD, starting from the sensation of itch, which evokes scratching behavior, thereby causing more damage to the defective skin barrier, which allows for the permeation of allergens and irritants, and the activation of alarm signals [50]. Previous studies demonstrated that itch was induced by multifaced pruritogens, including type 2 cytokines [51] (Figure 1).

### 4.1. IL-4 and IL-13

The presence of IL-4 receptor subunit α (IL-4Rα) on afferent neurons reinforces the potential of a relationship between the type 2 response and neural itch control. Oetjen et al. reported that the dorsal root ganglion (DRG) in mice and humans expressed IL-4Rα and IL-13Rα, and that IL-4 and IL-13 can directly activate sensory neurons. An injection of IL-4 enhanced the responsiveness of sensory neurons to many different pruritogens, such as histamine, chloroquine, and IL-31 via a signaling pathway that was dependent on IL-4Rα-Janus kinase (JAK), which led to the amplification of scratching behavior. Moreover, a treatment with a JAK inhibitor significantly attenuated recalcitrant chronic itch that was resistant to other immunosuppressive therapies [52]. The findings of clinical trials also supported the type 2 neuroimmune interaction by showing the responsiveness of itch to the inhibition of IL-4Rα by dupilumab and downstream JAK inhibition [53,54].

Previous studies reported prominent roles for IL-13 in AD, e.g., inflammation, skin barrier disruption, infection, itch, and epidermal thickening [2,55]. Elevated levels of IL-13 mRNA have been detected in both the lesional and non-lesional skin of AD patients [56], in addition to increases in the number of IL-13-producing circulating T cells [57], which were both closely associated with disease severity [55,56,57]. IL-13 has been suggested to drive inflammation in the periphery [55] and is considered to be pruritogenic on sensory neurons [52]. A low-dose (1 μg) intradermal injection of IL-13 induced scratching behavior in mice, while a combined exposure to IL-13 and IL-4 increased the frequency of scratching bouts, implicating IL-13 as the predominant acute pruritogen on peripheral sensory nerves [58]. On the other hand, Oetjen et al. demonstrated that a high-dose (2.5 μg) intradermal injection of IL-13 did not elicit acute itch in mice, suggesting that differences in IL-13 concentrations affect the scratching behavior in mice [52].

### 4.2. IL-31

Since the initial identification of the T cell-derived cytokine IL-31 in 2004 [59], AD patients were found to have elevated expression levels of IL-31 in skin-infiltrating cells (e.g., mononuclear cells) and IL-31 receptor subunit α (IL-31Rα) in keratinocytes and nerve fibers in the dermis [60]. IL-31Rα is mainly expressed in small- to medium-sized human DRG neurons, and is exclusively expressed by a subpopulation of TRPV1^+^/TRPA1^+^ DRG neurons [61]. In addition, several type 2 immune cells release IL-31 and induce itch through the direct stimulation of IL-31Rα [61]. 

Prolonged itch may be initiated by the overexpression of IL-31 and promotion of sensory neuronal outgrowth [62] and stimulation [63]. Transgenic IL-31 overexpression and subcutaneously administered IL-31 increased cutaneous nerve fiber density in lesional skin in vivo [62]. These findings suggest that the IL-31 axis plays an important role in the neuroimmune link between IL-31-expressing T cells and IL-31Rα-expressing sensory neurons [60,64], and may partly explain increased epidermal sensory nerve fiber density in AD patients [65,66,67] in the supreme “skin sensitivity” to minimal stimuli in AD patients.

However, the sequential in vivo imaging of peripheral sensory nerves and blood vessels in a mouse model of AD revealed that neural sprouting preceded vascularization, immune cell infiltration, and vascular permeability, suggesting that an allergic stimulation in chronic eczema requires neural recruitment and activation early in the process of the inflammatory cascade [65]. The development of early neuronal imprinting is followed by the recruitment of IL-31^+^ T cells to neuronal IL-31Rα^+^, and neuroimmune interactions may induce increases in epidermal nerve fiber density, inflammation, and itch [68]. 

Collectively, these findings indicate that IL-31 plays a central role in neuroimmune communication between Th2 cells (the main source of IL-31), sensory nerves, and keratinocytes, which are, in turn, involved in the pathophysiology of AD, including inflammation, epithelial disruption, and itch [68]. Furthermore, the attenuation of itch by nemolizumab, a humanized monoclonal anti-IL-31Rα antibody, supports the key role of IL-31 in AD-related itch [69].

### 4.3. IL-33

IL-33 is a member of the IL-1 cytokine family and is constitutively expressed in structural and lining cells exposed to the environment, including fibroblasts, the endothelium, keratinocytes, the gastrointestinal tract, and lungs. IL-33 activates allergic inflammation-related immune cells, such as basophils, mast cells, and macrophages as well as eosinophils and ILC2 (through its receptor ST2). Therefore, IL-33 plays a role in the mediation of type 2 immune responses [70,71,72,73,74,75].

IL-33 is one of the main mediators frequently associated with other cytokines. A stimulation with IL-33 was previously shown to augment the production of IL-5 and IL-13, which are constitutively expressed by fibrocytes [76]. In addition, the stimulation of human mast cells with IL-33 induced the expression of IL-31, which was augmented by neuropeptide substance P or IgE, in the presence or absence of IL-4 [77]. These findings suggest that neuroimmune interconnections between IL-33 and other cytokines may arise under allergic or inflammatory conditions.

Liu et al. detected the expression of ST2 on small- to medium-sized DRG neurons, including neurons that innervate the skin, in an urushiol-induced allergic contact dermatitis (ACD) mouse model. In the inflamed skin of this ACD mouse model, an increased level of IL-33 was responsible for the initiation of itch in sensitized mice. TRPV1 and TRPA1 ion channels mediated the activation of neurons by IL-33. Moreover, the blockade of IL-33/ST2 signaling attenuated the itch sensation in urushiol-challenged mice [78]. Although the comprehensive role of IL-33 in itch in AD remains unclear, these findings suggest that it plays an important role.

### 4.4. TSLP

Numerous studies suggest that TSLP produced by keratinocytes serves as a master switch that triggers both the initiation and maintenance of AD and the atopic march [79,80]. TSLP activates dendritic cells (DCs) to produce chemokines, which attract Th2 cells to the skin, which then produce proallergic cytokines, e.g., IL-4, IL-5, and IL-13. The up-regulated expression of TSLP has been reported in the skin of AD patients [81]. Wilson et al. showed that TSLP released from epidermal keratinocytes directly acted on cutaneous sensory neurons to initiate itch. They also found that an injection of TSLP bound to its receptor via the TRPA1 cation channel, which was expressed in neurons and promoted scratching behavior in mice. Therefore, the activation of primary afferent neurons and immune cells via the calcium-dependent TSLP release by keratinocytes may initiate skin inflammatory responses and induce itch signaling [82], such as in AD.

## 5. Treatment for Itch in AD

Despite numerous and extensive studies of the pathophysiology of itch in AD, currently available systemic treatments have limited potency and restricted use due to safety concerns. Newly emerging biologic agents may become superior AD treatments, and their efficacy and safety are now being investigated in systematic reviews and meta-analyses. At the time of writing, dupilumab was the only biologic therapy being extensively investigated, and although other drugs were promising, available data were insufficient. Longer follow-ups and larger population studies are required to obtain reliable biologic safety profiles [83]. Recently developed biologic agents related to type 2 inflammation for the treatment of itch in AD are summarized in Table 1.

### 5.1. Anti-IL-4 Receptor Antibody

A well-known human monoclonal antibody (mAb), dupilumab, binds to the shared alpha subunit of IL-4 and IL-13 receptors and induces the activation of T cells via the IL-4 and IL-13 pathways. This receptor has been detected on DCs, keratinocytes, and eosinophils [107]. The beneficial effects of dupilumab include the dose-dependent enhancement of the molecular signature in AD skin in vitro, and the down-regulated mRNA expression of the genes involved in activated T cells, DCs, or eosinophils [108]. In clinical trials on dupilumab, clinical symptoms were ameliorated in adult patients with moderate-to-severe AD [53,84]. Two large phase-3 trials (SOLO1 and 2) demonstrated that in comparisons with controls, dupilumab attenuated the signs and symptoms of AD, including improvements in the Numerical Rating Scale (NRS) for itch by at least 4 points, anxiety, depression, and QoL [85,109].

The effects of dupilumab in real-world patient populations were consistent with the findings of clinical trials [110,111]. The adverse events (AEs) of dupilumab are minimal and tolerable, with ocular side effects (particularly conjunctivitis) being the most common [110,111,112]. Furthermore, current trial data show the minimal need for laboratory monitoring during consumption. An open-label extension study of adults with AD treated weekly with dupilumab for 72 weeks reported continuing efficacy with no additional safety effects; however, longer observations for AEs are advised [86,113]. Dupilumab was the first biologic to be approved by the US Food and Drug Administration (FDA) as the first-line treatment for moderate-to-severe AD in patients aged 6 years and older in the USA and it has also been approved for use in patients aged 12 years and older in the EU [4,107].

The mechanism of action of dupilumab does not only involve the IL-4/IL-13 pathways. Mack et al. performed the high-dimensional immune profiling of patients with AD and found deficiencies in specific subsets of natural killer (NK) cells. NK cell defects were reversed after the blockade of type 2 cytokines in patients with AD. A treatment with dupilumab was associated with the significant recovery of NK cells, as confirmed by clinical flow cytometry, together with improved clinical scores and inflammatory cytokine levels. These findings suggest that NK cells play an immunoregulatory role in type 2 inflammation in AD, possibly via the IL-4 pathway [114].

### 5.2. Anti-IL-13

Anti-IL-13 interrupts type 2 immune signaling by directly binding to soluble IL-13 [1,115]. Agents for anti-IL-13 activity include lebrikizumab, which selectively hinders the establishment of the IL-13Rα1/IL-4Rα heterodimer receptor signaling complex [87], and tralokinumab, which specifically binds to IL-13, thereby preventing any interplay with the IL-13 receptor and subsequent downstream IL-13 signaling [88].

A phase 2b placebo-controlled randomized clinical trial (RCT) on patients with moderate-to-severe AD demonstrated that a 16-week treatment with lebrikizumab significantly improved pruritus NRS by ≥4 points, clinical scores, and QoL in a dose-dependent manner with good safety [87]. In two parallel 16-week phase 3 (ECZTRA1 and 2) trials on moderate-to-severe AD adults, tralokinumab monotherapy was more effective than a control treatment after 16 weeks (improvement in pruritus NRS by ≥4 points, sleep interference, QoL, and clinical signs), and was tolerated well at 52 weeks [89]. An additional phase 3 (ECZTRA3) trial on these patients demonstrated that the combination of tralokinumab and topical corticosteroids (TCS) as needed was effective and achieved similar favorable outcomes and AEs to those in ECZTRA1 and 2 [88].

### 5.3. Anti-IL-31 Signaling

#### 5.3.1. Anti-IL-31

An agent targeting IL-31 for clinical use (BMS-981164) was examined in a phase I study between 2012 and 2015 [116]; however, the findings obtained were not released until now (https://clinicaltrials.gov/ct2/show/NCT01614756, accessed on 15 September 2021).

#### 5.3.2. Anti-IL-31RA

Nemolizumab is a subcutaneously administered humanized mAb against IL-31Rα, which is involved in itch in AD [116]. Among IL-31 strategies to alleviate pruritus, only nemolizumab has successfully completed late-stage clinical studies. This drug binds to IL-31Rα in cells such as neurons, blocking the binding of IL-31, which inhibits IL-31 signaling [107]. Moreover, nemolizumab has been investigated for the refinement of sleep, daily functioning, and QoL disruptions in patients with AD [90].

In an RCT, double-blind phase I/Ib study, the administration of nemolizumab as a single subcutaneous dose improved the pruritus visual analog score (VAS) score to approximately 50% by week 4, in contrast to 20% by a control treatment. It improved sleep comfort and decreased the need to use hydrocortisone butyrate. Furthermore, there were no serious AEs or discontinuation due to AEs [91].

In a phase 2 trial, nemolizumab significantly improved the pruritus VAS score (43.7%) vs. control (20.9%), which was inadequately controlled by topical treatments in moderate-to-severe AD patients. The incidence and types of AEs in the nemolizumab group were similar to those in the placebo group, except for exacerbations in AD and peripheral edema, which were more prevalent in those receiving nemolizumab [69]. In a phase 2B 24-week RCT study, nemolizumab achieved improvements in pruritus NRS by ≥4 points, the NRS-sleep scale, Investigator Global Assessment (IGA) response, EASI score, and SCORAD [92].

In a 16-week double-blind phase 3 trial, moderate-to-severe pruritus AD patients with an inadequate response to topical agents showed greater improvements in the pruritus VAS score with the subcutaneous administration of nemolizumab plus topical agents (42.8%) than with placebo plus topical agents (21.4%). Injection-site reactions were more common in the nemolizumab group than in the placebo group. Longer and larger trials to establish the long-lasting impact and safety of nemolizumab for AD are needed [90].

### 5.4. JAK Inhibitors

The JAK and signal transducer and activator of transcription (JAK-STAT) pathway is used by cytokines as an intracellular signaling pathway. The phosphorylation, dimerization, and translocation of specific STAT proteins occur in the nucleus after the activation of JAK proteins, and each JAK protein then communicates with numerous cytokine receptors involved in inflammatory diseases [117]. The JAK-STAT pathway has been reported to encompass several tyrosine kinase proteins that interact with the common γ-chain of cytokine receptors and generate cytokine-mediated responses, and is essential for T helper 2 cell differentiation [107,118].

Baricitinib, an oral selective JAK1/JAK2 inhibitor, was the first oral JAK inhibitor to progress to phase 3 clinical trials for AD [119]. In two multicenter, double-blind, phase III monotherapy trials (BREEZE-AD1 and BREEZE-AD2) on moderate-to-severe AD adults, baricitinib attenuated the clinical signs of AD within 16 weeks with the prompt amelioration of itch. AEs were similar between the baricitinib and control groups [93]. In another phase 3 RCT (BREEZE-AD7), moderate-to-severe AD adults with an inadequate response to TCS therapy who received 4 mg of baricitinib plus TCS showed significant improvements in pruritus NRS by ≥4 points, the signs and symptoms of AD, sleep, skin pain, and QoL. The safety profile was similar to that reported in previous studies on baricitinib for AD [94]. Baricitinib has been approved for AD in Japan and the EU, and is being investigated in phase 3 trials in other countries (https://clinicaltrials.gov/ct2/results?cond=Atopic+Dermatitis&term=baricitinib&cntry=&state=&city=&dist=, accessed on 15 September 2021).

Delgocitinib (formerly JTE-052) is a novel, small-molecule JAK inhibitor that is being developed in Japan. It exerts inhibitory effects on JAK1, JAK2, JAK3, and tyrosine kinase 2 [120]. In a phase 3 RCT, double-blind open-label study, 0.5% delgocitinib ointment improved pruritus NRS points (daytime and nighttime) as well as clinical signs and symptoms with good safety for up to 28 weeks in Japanese adults with moderate-to-severe AD [95]. A long-term study of the safety and efficacy of this ointment revealed that it was tolerated well and effectively improved pruritus NRS points up to 52 weeks [96]. Delgocitinib has been approved for the treatment of AD in Japan. It is being investigated in phase 3 trials elsewhere (https://clinicaltrials.gov/ct2/show/NCT04949841?term=delgocitinib&cond=Atopic+Dermatitis&draw=2&rank=6, accessed on 15 September 2021).

Tofacitinib citrate, an oral small-molecule JAK1/3 inhibitor that was initially approved to treat rheumatoid arthritis, acts by blocking Th2 cytokine signaling (IL-4, -5, and -13). Tofacitinib is presently being examined for its potential as a treatment for AD [107]. The efficacy of topical tofacitinib was evaluated in 69 adults with mild-to-moderate AD in a phase 2a, double-blind RCT. Tofacitinib 2% ointment showed significantly higher efficacy than a control treatment for improvements in the Itch Severity Item score and clinical signs, with the early onset of effects and tolerable AEs [54].

Oral selective JAK1 inhibitors, such as abrocitinib and upadacitinib, have been shown to alleviate itch and clinical manifestations in patients with moderate-to-severe AD. Two phase 3 RCTs demonstrated that abrocitinib monotherapy for 12 weeks was effective and tolerated well, e.g., improvements in pruritus NRS by ≥4 points, EASI, and IGA responses [97,98]. Upadacitinib has been approved for moderate-to-severe active rheumatoid arthritis, and may disrupt JAK1 signaling followed by the Th2 cytokines involved, thereby alleviating chronic itch [99,100]. In a phase 2B dose-ranging RCT, 30 mg of upadacitinib was shown to improve pruritus NRS by ≥4 points as well as clinical manifestations [99]. The combination of upadacitinib and TCS in a phase 3 double-blind AD study achieved similar clinical outcomes [100] and was tolerated well [101].

### 5.5. A Phosphodiesterase 4 (PDE4) Inhibitor

PDE4 inhibitors decrease cyclic adenosine monophosphate concentrations, which reduces the production of proinflammatory cytokines involved in AD. Crisaborole 2% ointment was the first nonsteroidal PDE4 inhibitor used to treat mild-to-moderate AD [1]. Two pivotal phase 3 28-day, double-blind RCTs of crisaborole 2% in mild-to-moderate AD adults showed the earlier achievement and greater proportion of itch improvements (measured by the severity of pruritus scale and IGA scores) [102]. Moreover, a post hoc analysis revealed the significantly earlier achievement of itch management by crisaborole than by a control treatment [103].

Another study reported that crisaborole reversed the biomarker profiles of skin inflammation (e.g., Th2 and Th17/Th22 axes) and improved barrier function (e.g., immune cell infiltration and epidermal hyperplasia/proliferation) with good clinical efficacy (pruritus NRS and clinical signs), thereby supporting the therapeutic benefits of targeting PDE4 in AD patients [104]. Crisaborole 2% ointment was approved by the FDA for the treatment of mild-to-moderate AD in infants aged 3 months and older.

### 5.6. Anti-TSLP

Tezepelumab (AMG 157) is a human anti-TSLP monoclonal immunoglobulin G2λ that specifically binds to human TSLP and inhibits interactions with its receptor [121]. In a double-blind, placebo-controlled study, a treatment with tezepelumab attenuated allergen-induced bronchoconstriction and indexes of airway inflammation before and after an allergen challenge in mild allergic asthma patients [121]. A phase 2 clinical trial conducted among patients receiving long-acting beta-agonists and medium-to-high doses of inhaled glucocorticoids showed lower rates of clinical asthma exacerbation by tezepelumab than by a placebo. The incidence of AEs was similar among trial groups [122].

A phase 2a study on tezepelumab- or placebo plus TCS-treated moderate-to-severe AD adults reported slight improvements (clinical signs and pruritus) from the control following 12 weeks of treatment, and greater responses at 16 weeks. In tezepelumab vs. placebo groups, pruritus NRS were 33.54 vs. 25.41 (*p* = 0.258), EASI50 responses were 64.7% vs. 48.2% (*p* = 0.091), and SCORAD50 were 41% vs. 29.4% (*p* = 0.219), respectively [105]. Overall, these findings suggest that targeting TSLP is beneficial for the treatment of asthma, but may not be as effective at attenuating dermatitis-related itch.

### 5.7. Anti-IL-33

A previous study evaluated the efficacy of vaccination against IL-33 in a house dust mite (HDM)-induced airway inflammation mouse model. The inhibition of HDM-induced airway hyperresponsiveness and inflammation and the production of inflammatory cytokines were observed after the vaccination against IL-33 [123]. In a 6-week placebo-controlled phase 2a study, a single dose of etokimab, an anti–IL-33 biologic, was administered to desensitize peanut-allergic adults. The findings obtained revealed the safety of etokimab, and that a single dose of etokimab may desensitize peanut-allergic individuals and attenuate atopy-related AEs [124]. Chen et al. investigated the efficacy of etokimab in a proof-of-concept clinical study among moderate-to-severe AD. A single intravenous dose of 300 mg of etokimab achieved improvements in 5D itch scores, EASI, SCORAD, IGA, and DLQI 29 days after drug administration and was generally tolerated [106]. The inhibition of IL-33 appears to be effective for alleviating allergic disease symptoms, including AD; however, further studies on its efficacy are needed.

## 6. Conclusions

The pathogenesis of AD encompasses various immune pathways. Recent studies revealed that type 2 immune inflammation is the dominant pathway involved, driven by innate type 2 ILC and Th2 cells as well as their cytokines, such as IL-4 and IL-13. Itch is a sensation associated with AD. Previous studies revealed that neuroimmune communication is a key player in the development of itch in inflammatory skin diseases, such as AD. Therefore, targeting type 2 pathways in the neuroimmune interaction appears to be a reasonable therapeutic strategy for itch in AD. Recently developed biologic agents targeting type 2-associated cytokines have achieved promising outcomes. The mAb anti-IL-4Rα (dupilumab) and topical PDE4 inhibitor (crisaborole) have been approved by the FDA for moderate-to-severe and mild-to-moderate AD, while JAK inhibitors (baricitinib and delgocitinib) have been approved for AD in Japan. Based on the findings of recent clinical trials on the treatment of itch in AD, dupilumab appears to be the best option for moderate-to-severe AD, and crisaborole 2% for mild-to moderate AD. Further studies on other agents will offer novel insights into the underlying pathogeneses and new targeted treatment alternatives for itch in AD.

## Figures and Tables

**Table 1:** Therapeutic potential of the biologic agent-type 2 inflammation-related regulation of itch in atopic dermatitis.

Mediator	Mechanism	Drug	Status	Clinical Effects	References
IL-4, IL-13	Anti-IL-4Rα	Dupilumab	Approved for moderate-to-severe AD (FDA)	Improvement in pruritus NRS by ≥4 points; IGA, EASI, SCORAD, DLQI	[53,84,85,86]
IL-13	Anti-IL-13	Lebrikizumab	Phase 2b	Improvement in pruritus NRS by ≥4 points; EASI, IGA, BSA, POEM	[87]
		Tralokinumab	Phase 3	Improvement in pruritus NRS by ≥4 points; IGA, BSA, EASI, SCORAD, POEM	[88,89]
IL-31	Anti-IL-31	BMS-981164	Phase 1	Data not yet released	https://clinicaltrials.gov/ct2/show/NCT01614756 (accessed on 15 September 2021)
	Anti-IL-31Rα	Nemolizumab	Phase 3	Improvement in pruritus VAS by 40–60%	[69,90,91,92]
JAK	JAK1/JAK2 inhibitor	Baricitinib	Approved for AD in Japan and the EU; undergoing phase 3 trials in other countries	Improvement in pruritus NRS by ≥4 points; IGA, EASI, SCORAD, skin pain, POEM, DLQI	[93,94] https://clinicaltrials.gov/ct2/results?cond=Atopic+Dermatitis&term=baricitinib&cntry=&state=&city=&dist= (accessed on 15 September 2021)
	JAK1, JAK2, JAK3, and a tyrosine kinase 2 inhibitor	Delgocitinib 0.5% (topical)	Approved for AD in Japan; undergoing phase 3 trials in other countries	Improvement in pruritus NRS points; IGA, EASI, BSA	[95,96] https://clinicaltrials.gov/ct2/show/NCT04949841?term=delgocitinib&cond=Atopic+Dermatitis&draw=2&rank=6 (accessed on 15 September 2021)
	JAK1/3 inhibitor	Tofacitinib 2% (topical)	Phase 2a	Improvement in ISI; EASI, PGA, BSA	[54]
	JAK1 inhibitor	Abrocitinib (oral)	Phase 3	Improvement in pruritus NRS by ≥4 points; IGA, EASI	[97,98]
		Upadacitinib (oral)	Phase 3	Improvement in pruritus NRS by ≥4 points; IGA, EASI	[99,100,101]
PDE4	PDE4 inhibitor	Crisaborole 2% (topical)	Approved for mild-to-moderate AD (FDA)	Improvement in the severity pruritus scale & NRS points; IGA, AD signs, DLQI	[102,103,104]
TSLP	Anti-TSLPR	Tezepelumab	Phase 2a	Improvement in pruritus NRS points & the 5-D itch scale; EASI, IGA, SCORAD (Numerical improvement *)	[105]
^#^IL-33	Anti-IL-33	Etokimab	Phase 2a proof-of-concept study	Improvement in 5D itch scores; EASI, SCORAD, IGA, DLQI	[106]

* No significant difference; ^#^ Proof-of-concept study; NRS = Numerical Rating Scale; FDA = Food and Drug Administration; IGA = Investigator Global Assessment; EASI = Eczema Area and Severity Index; SCORAD= SCORing Atopic Dermatitis; DLQI= Dermatology Life Quality Index; VAS = Visual Analog Score; ISI = Itch Severity Item; BSA = Body Surface Area; POEM = Patient-Oriented Eczema Measure.
